# Bioassays to Monitor Taspase1 Function for the Identification of Pharmacogenetic Inhibitors

**DOI:** 10.1371/journal.pone.0018253

**Published:** 2011-05-25

**Authors:** Shirley K. Knauer, Verena Fetz, Jens Rabenstein, Sandra Friedl, Bettina Hofmann, Samaneh Sabiani, Elisabeth Schröder, Lena Kunst, Eugen Proschak, Eckhard Thines, Thomas Kindler, Gisbert Schneider, Rolf Marschalek, Roland H. Stauber, Carolin Bier

**Affiliations:** 1 Institute for Molecular Biology, Centre for Medical Biotechnology (ZMB), University Duisburg-Essen, Essen, Germany; 2 Mainzer Screening Center (MSC), University Medical Center of the Johannes Gutenberg-University of Mainz, Mainz, Germany; 3 Institute of Pharmaceutical Biology/ZAFES, Goethe-University, Frankfurt/Main, Germany; 4 Institute Organic Chemistry and Chemical Biology/ZAFES, Goethe-University, Frankfurt/Main, Germany; 5 Institute of Biotechnology and Drug Research Kaiserslautern (IBWF), Kaiserslautern, Germany; 6 Department of Hematology/Oncology, University Medical Center of the Johannes Gutenberg-University of Mainz, Mainz, Germany; University of Minnesota, United States of America

## Abstract

**Background:**

Threonine Aspartase 1 (Taspase1) mediates cleavage of the mixed lineage leukemia (MLL) protein and leukemia provoking MLL-fusions. In contrast to other proteases, the understanding of Taspase1's (patho)biological relevance and function is limited, since neither small molecule inhibitors nor cell based functional assays for Taspase1 are currently available.

**Methodology/Findings:**

Efficient cell-based assays to probe Taspase1 function *in vivo* are presented here. These are composed of glutathione S-transferase, autofluorescent protein variants, Taspase1 cleavage sites and rational combinations of nuclear import and export signals. The biosensors localize predominantly to the cytoplasm, whereas expression of biologically active Taspase1 but not of inactive Taspase1 mutants or of the protease Caspase3 triggers their proteolytic cleavage and nuclear accumulation. Compared to *in vitro* assays using recombinant components the *in vivo* assay was highly efficient. Employing an optimized nuclear translocation algorithm, the triple-color assay could be adapted to a high-throughput microscopy platform (Z'factor = 0.63). Automated high-content data analysis was used to screen a focused compound library, selected by an *in silico* pharmacophor screening approach, as well as a collection of fungal extracts. Screening identified two compounds, N-[2-[(4-amino-6-oxo-3H-pyrimidin-2-yl)sulfanyl]ethyl]benzenesulfonamide and 2-benzyltriazole-4,5-dicarboxylic acid, which partially inhibited Taspase1 cleavage in living cells. Additionally, the assay was exploited to probe endogenous Taspase1 in solid tumor cell models and to identify an improved consensus sequence for efficient Taspase1 cleavage. This allowed the *in silico* identification of novel putative Taspase1 targets. Those include the FERM Domain-Containing Protein 4B, the Tyrosine-Protein Phosphatase Zeta, and DNA Polymerase Zeta. Cleavage site recognition and proteolytic processing of these substrates were verified in the context of the biosensor.

**Conclusions:**

The assay not only allows to genetically probe Taspase1 structure function *in vivo*, but is also applicable for high-content screening to identify Taspase1 inhibitors. Such tools will provide novel insights into Taspase1's function and its potential therapeutic relevance.

## Introduction

Besides their critical role in intra- and intercellular “waste management”, proteases are currently accepted as important signaling molecules involved in numerous biological and pathological functions [1], [2]. These include metabolism, tissue remodeling, apoptosis, cell proliferation and migration [1], [3]. Thus, protease signaling needs to be strictly regulated, and the deregulation of protease activity may contribute to various pathologies, including neoplastic diseases [1], [2], [4].

The human *Threonine Aspartase 1/Taspase1* gene encodes a protein of 420 amino acids (aa), representing the proenzyme of the protease. In contrast to the other exclusively *cis*-active type 2 Asparaginases, only Taspase1 is also able to cleave other substrates in *trans* [5]. Therefore, Taspase1 represents a distinct class of proteolytic enzymes. Taspase1 mediates cleavage of proteins by recognizing a conserved peptide motif with an aspartate at the P^1^ position [5]. The N-terminal threonine (Thr^234^) is generated by autoproteolysis of the Taspase1 proenzyme (*cis*-cleavage) into the two subunits α and β, which appear to assemble into an asymmetric 28 kDa/22 kDa α_2_/β_2_-heterotetramer, the active protease [6]. The discovery of Taspase1 founded a new class of endopeptidases that utilize the N-terminal threonine of its mature β-subunit as the active site [5]. Mutation of this catalytic nucleophile, Thr^234^, abolishes Taspase1's proteolytic activity [5], [6].

Taspase1 was first identified as the protease responsible for cleavage of the Mixed Lineage Leukemia (MLL) protein at conserved (Q^3^X^2^D^1^↓G^1′^) sites [5]. Proteolytic cleavage of MLL is considered to stabilize the MLL protein [7], [8] as a crucial event for proper *Hox* gene expression and normal cell cycle [9], [10]. However, *MLL* is also found as a translocation partner in a variety of acute leukemias [5], [9], [10], [11], [12]. Interestingly, we recently showed that only AF4•MLL but not the reciprocal translocation product, MLL•AF4, lacking the Taspase1 cleavage site, can cause proB ALL in a murine model [13].

Thus, proteolytic cleavage of MLL-fusion proteins by Taspase1 is considered a critical step for MLL-mediated tumorigenesis, although the molecular details are not yet resolved [5], [9], [10], [11], [12].

Besides Taspase1's role in leukemogenesis the protease was suggested to be also overexpressed solid tumors [10]. In this respect, recent data indicate that also other regulatory proteins, such as the precursor of the Transcription Factor IIA (TFIIA) or Drosophila HCF [7], [14], are Taspase1 targets. Hence, there is an increasing interest in defining novel Taspase1 targets. However, the molecular mechanisms how Taspase1 affects biological functions through site-specific proteolysis of its substrates and what other cellular programs are regulated by Taspase1's degradome under normal or pathophysiological conditions is completely unknown.

Besides genetic instruments, chemical decoys allowing the targeted inhibition/activation of proteins are powerful tools to dissect complex biological pathways. Small molecules that allow a chemical knock out of a cellular reaction or a cell phenotype can be selected by phenotypic screens, and used as molecular tools to identify previously uncharacterized proteins and/or molecular mechanisms. Hence, chemogenomics as studying the interaction of biological systems with exogenous small molecules, i.e., analyzing the intersection of biological and chemical spaces [15], [16], seems an attractive approach to also dissect Taspase1 functions. Unfortunately, Taspase1's catalytic activity is not affected by common protease inhibitors and no small molecule inhibitors for this enzyme are currently available to dissect Taspase1's function *in vivo* [5], [17].

As biochemical data or potential drugs must be effective at the cellular level, reliable cell-based assays (CBA) for Taspase1 are urgently needed. Often, redistribution approaches, as cell-based assay technology that uses protein translocation as the primary readout have been used to study the activity of cellular signaling pathways [18], [19]. Protein targets are labeled with autofluorescent proteins and are read using high-throughput, microscope-based instruments [18], [19]. Although, protein translocation assays have the potential for high-content (HCS), high-throughput screening (HTS) applications, such assays are generally not used for proteases.

Here, the spatial and functional division into the nucleus and the cytoplasm was exploited to design a translocation-based Taspase1-biosensor assay. The CBA was adapted on a HTS platform, employed to identify potential Taspase1 small molecule inhibitors, and was used to study Taspase1 function in living cells.

## Results

### Assays to study Taspase1 cleavage activity

To characterize the sequence and spatial requirements for efficient Taspase1 processing as well as to screen for potential Taspase1 inhibitors, we first tested an *in vitro* cleavage assay (Suppl. Figure S1B). Attempts to express and purify Taspase1 under native conditions as a GST-Taspase1-GFP fusion failed due to extensive protein aggregation, which was evident already in bacteria (Suppl. Figure S1A). Therefore, His-tagged Taspase1 (rTasp1) was purified by imidazol and nickel chelating affinity chromatography. Incubation of the substrate, GST-2Cl, containing the MLL cleavage sites 1 and 2 (MLL aa 2650–2808), with increasing amounts of rTasp1 resulted in the proteolytic cleavage of the substrate as well as in the autocatalytic processing of the proenzyme. However, cleavage occurred slowly, and a high ratio of enzyme/substrate was required for complete substrate cleavage (Suppl. Figure S1C and S1D). These results indicated the possibility that bacterially expressed Taspase1 displays only an attenuated catalytic activity.

**Figure 1:**
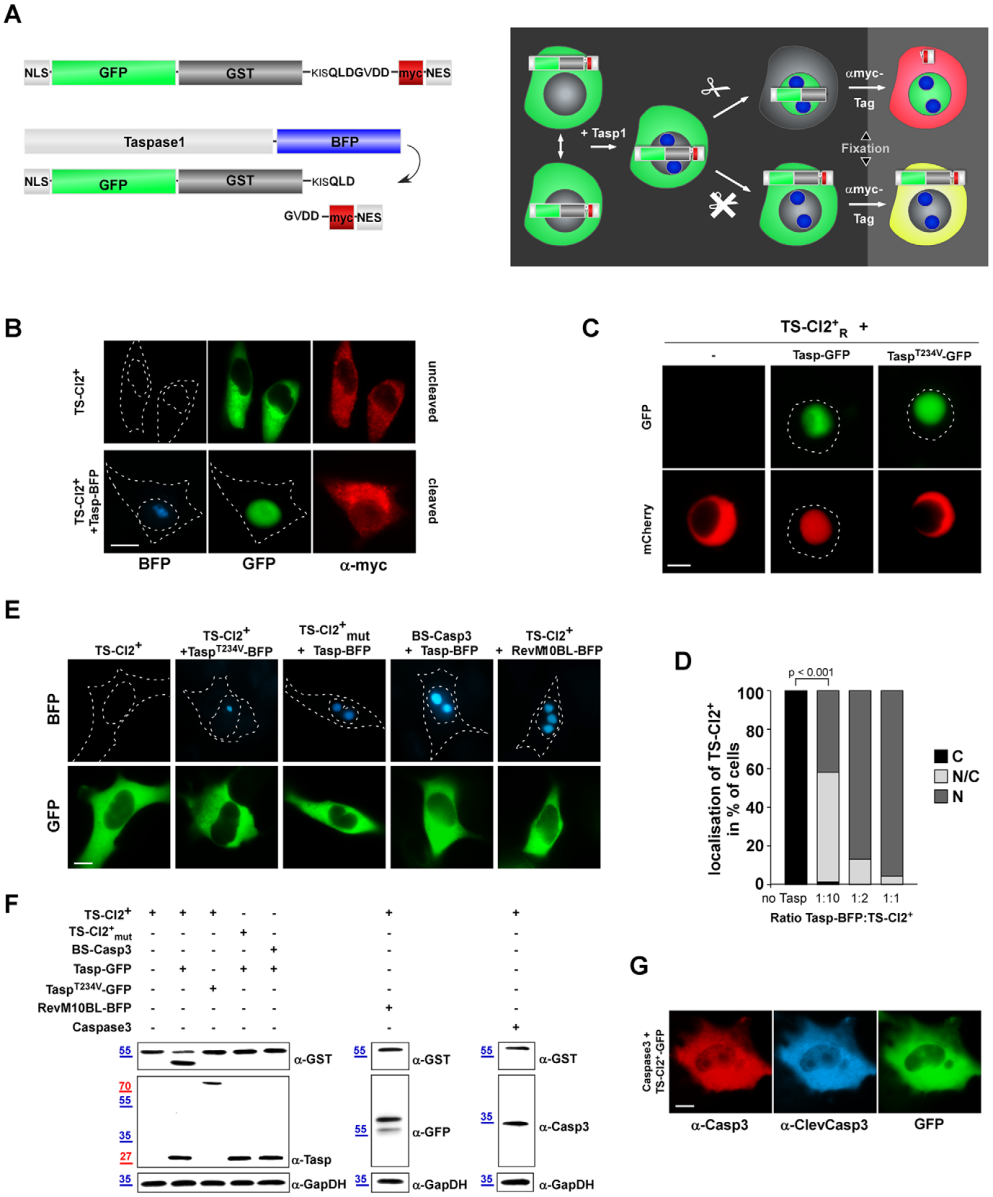
Multi-color translocation biosensor assays to analyse Taspase1-mediated cleavage in living cells. **A.** *In vivo* assay. Schematic domain organization and principle of the *TS-Cl2^+^* translocation sensor to probe Taspase1 activity. *TS-Cl2^+^* is composed of GST, GFP, combinations of a nuclear import (NLS) and a Myc epitope-tagged export (NES) signal, combined with the Taspase1 cleavage site from MLL (Cl2^+^; aa ^2713^KISQLDGVDD^2722^). *TS-Cl2^+^* localizes predominantly to the cytoplasm but is continuously shuttling between the nucleus and the cytoplasm. Co-expression of Taspase1-BFP (Tasp-BFP) results in the loss of the NES by proteolytic cleavage of the biosensor, triggering nuclear accumulation of the green fluorescent indicator protein. **B.** Expression of Taspase1 results in nuclear translocation of *TS-Cl2^+^* in living HeLa transfectants. The autofluorescent biosensor is predominantly cytoplasmic (upper panel), whereas co-expression of Taspase1-BFP results in its proteolytic cleavage and nuclear accumulation (lower panel). Myc-NES_Rev_ was detected by indirect immunofluorescence using an α-myc-tag Ab. **C.** The translocation biosensor is functional not only in adherent but also in leukemia cell models. K562 cells were transfected with expression plasmids encoding the indicated proteins. Coexpression of Tasp-GFP but not of inactive Tasp^T234V^-GFP resulted in proteolytic cleavage and nuclear accumulation of the red fluorescent biosensor, *TS-Cl2^+^_R_*. Biosensor localization was analyzed 48 h post transfection in at least 200 fluorescent living cells, and representative images are shown. Dashed lines mark nuclear/cytoplasmic cell boundaries obtained from the corresponding phase contrast images. **D.** Quantitation of TS-Cl2^+^ processing *in vivo*. HeLa cells were transfected with the indicated ratios of enzyme (Taspase1-BFP) and substrate (TS-Cl2^+^). 48 h later, the percentage of cells showing cytoplasmic (C), cytoplasmic and nuclear (N/C) or nuclear (N) fluorescence was determined for at least 200 fluorescent cells. Cleavage-induced nuclear accumulation of the biosensor significantly increased already at a ratio of 1/10 (***: p<0.0001). Results from a representative experiment are shown. **E.** Biosensor assay specificity. Biosensors containing a non-functional Taspase1 cleavage site (*TS-Cl2^+^_mut_*) or a cleavage site for Caspase3 (*BS-Casp3*) remained cytoplasmic upon co-expression of Tasp-BFP. No nuclear accumulation of *TS-Cl2^+^* was observed upon coexpression of inactive Tasp^T234V^-BFP or the nucleolar RevM10BL-BFP protein. **F.** Cleavage of *TS-Cl2^+^, TS-Cl2^+^_mut_ or BS-Casp3* analyzed by immunoblot. 293T cells were transfected with the indicated biosensors together with the indicated Taspase1 expression plasmids, the empty vector (control), RevM10BL-BFP or the protease Caspase3. Expression of proteins and cleavage products in cell lysates was visualized using α-GST, -Taspase1, -GFP or -Casp3 Abs. GAPDH served as loading control. **G.** Ectopic expression of Caspase3 does not induce cleavage and nuclear translocation of *TS-Cl2^+^*. Caspase3 expression was visualized by IF using α-Casp3 antibody, its activation by α-ClevCasp3 Ab. GFP/BFP were visualized by fluorescence microscopy. Scale bars, 10 µm.

To circumvent the limitations of the *in vitro* assay, we hence focused on the most relevant test tube, the living cell. As shown in our previous studies, translocation-based autofluorescent biosensors are powerful tools to assess protein-protein interaction as well as nucleo-cytoplasmic transport *in vivo* [20], [21]. To generate a Taspase1 biosensor, we integrated the Taspase1 cleavage site from MLL (CS2; aa ^2713^KISQLDGVDD^2722^) into a biosensor backbone, composed of GST, GFP, the SV40 large T-antigen nuclear import signal (NLS) and a Myc-epitope-tagged nuclear export signal from the HIV-1 Rev protein (NES_Rev_) (Figure 1A). The rationale of this specific modular set-up was that Taspase1-mediated cleavage of the biosensor should liberate the NES_Rev_ triggering nuclear accumulation of the fluorescent indicator protein. Indeed, the resulting NLS-GFP/GST-CS2-NES_Rev_ fusion protein (*TS-Cl2^+^*) localizes predominantly to the cytoplasm (Figure 1B), since the NES activity is dominant over the NLS. Though, *TS-Cl2^+^* is continuously shuttling between the nucleus and the cytoplasm, as confirmed by treatment with the export inhibitor LeptomycinB (LMB), which abrogates nuclear export leading to nuclear accumulation of the biosensor (Suppl. Figure S2C). Similar results were obtained for a biosensor containing the red fluorescent protein, mCherry (mCh), instead of GFP (NLS-mCh/GST-CS_T_-NES_Rev_ = *TS-Cl2^+^_R_*) (Figure 1C).

**Figure 2:**
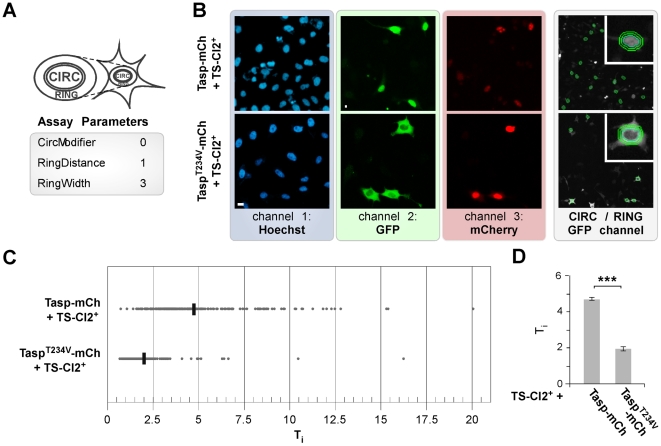
Biosensor assay adaptation onto a HTS platform. **A.** Object selection parameters for nuclear (CIRC) and cytoplasmatic compartment (RING) analysis. **B.** Biosensor translocation assay analysis using the Cellomics Arrayscan® VTI platform. HeLa transfectants coexpressing TS-Cl2^+^ and active or inactive (Tasp^T234V^) mCherry fusions were fixed 48 h post transfection and nuclei marked by Hoechst 33342. The Hoechst 33342, GFP, and mCherry signals were recorded in channels 1, 2 and 3, respectively. Overlay with the CIRC mask and RING region is outlined for GFP (right panel). Representative images are shown. Scale bar, 10 µm. **C.** Translocation index (T_i_ = CIRC∶RING) plotted for coexpression of Tasp1-mCh variants on a single cell basis. Values were derived from analyzing ∼400 cells/well. Mean from three wells is indicated. **D.** T_i_ was highly significantly increased upon coexpression of active compared to inactive Taspase1 (***: p<0.0001). Columns, mean; bars, SD.

Importantly, cotransfection of either the nuclear/nucleolar Taspase1-BFP (Tasp-BFP) or -mCherry (Tasp-mCh) results in the proteolytic cleavage of Myc-RevNES and the subsequent nuclear accumulation of *TS-Cl2^+^* in various epithelial and liquid cancer cell lines (Figure 1B/C and Figure 2B). Cleavage which was already evident 24 h post transfection and similar results were obtained 48 h post transfection (data not shown). As a control, a construct containing a non-functional Taspase1 cleavage site (*TS-Cl2^+^*_mut;_ aa ^2713^KISQL**AA**VDD^2722^) or a cleavage site for Caspase3 (*BS-Casp3*; aa KRKGDEVDGVDE) remained cytoplasmic not only under identical experimental conditions (Figure 1E), but also when cells were observed after 48 h (data not shown). Also, no nuclear accumulation was observed upon coexpression of the catalytically inactive Tasp^T234V^-BFP fusion, in which Thr^234^ was changed into Val (Tasp^T234V^) [5], [6] or of the nucleolar HIV-1 Rev-BFP protein, underpinning the assay's specificity (Figure 1E). Proteolytic processing of the biosensor upon expression of untagged or tagged Taspase1 was independently confirmed by immunoblot analysis (Figure 1F and data not shown). Even coexpression of an unrelated protease, such as Caspase-3 or -9, did not affect the cytoplasmic localization of the biosensor (Figure 1G and data not shown).

Notably, in contrast to the high amounts of rTasp1 required for cleavage *in vitro* (enzyme/substrate = 1∶2), cotransfection of enzyme (Taspase1-BFP) and substrate (TS-Cl2^+^) expression plasmid even at a ratio of 1∶10, was sufficient to catalyze efficient cleavage and nuclear accumulation of the biosensor (Figure 1D).

Collectively, the results clearly underlined the practical advantages and biological relevance of the cellular assay to search for pharmacogenomic Taspase1 inhibitors.

### Triple-color biosensor-based high content screening for Taspase1 inhibitors

The robust performance of the *TS-Cl2^+^* CBA met critical requirements for high content screening: the biosensor was non-toxic, localized to the cytoplasm in the absence of ectopically expressed Taspase1, and efficiently accumulated in the nucleus following Taspase1-specific cleavage. Hence, we tested whether the assay can also be used on a high-throughput microscopy based screening platform.

As cell lines inducibly expressing biosensors may facilitate certain HCS/HTS applications, we generated stable Tet-off *TS-Cl2^+^_TRE_* cell lines (Suppl. Figure S2A/B). The tetracycline (doxycycline)-regulated system has been used successfully in various applications [22]. Whereas expression of *TS-Cl2^+^_TRE_* was blocked in the presence of doxycycline (Dox), Dox removal induced *TS-Cl2^+^_TRE_* expression (Suppl. Figure S2B). Cleavage of *TS-Cl2^+^_TRE_* by the endogenous Taspase1 subsequently resulted in nuclear accumulation of the biosensor (Suppl. Figure S2B). Although this cell system circumvents the need for cotransfection of Taspase1, the levels of *TS-Cl2^+^_TRE_* are low compared to transient expression, which we considered as a potential limitation for HTS applications.

Thus, we decided to use transiently expressing transfectants for the HTS assay. First, due to the low quantum yield of BFP, the high cellular and sometimes observed compound autofluorescence background upon UV-excitation, a green/red fluorescence dual-color screening assay was developed. Therefore, BFP was replaced by mCherry in the Taspase1 expression plasmids (Tasp-mCh). Similar to GFP fusions, Tasp-mCh was biologically fully active, whereas the inactive mutant Tasp^T234V^-mCh did not cleave the GFP-based biosensor (Figure 2B) even after 48 h.

Second, for HCS the assay was adapted to the Cellomics Arrayscan®VTI platform. For this purpose, the molecular translocation assay was adjusted by modifying several parameters to ensure optimal object identification, including the adjustment of the background correction and to define the threshold of pixels derived from the Hoechst 33342 signal (Figure 2A). This calculation resulted in an optimized object identification, capable to automatically excluding “non cellular” irregular objects (too small/big, debris, compound aggregates, etc.) in channel 1. By adjusting object segmentation parameters, the fitting of the nuclear mask to the Hoechst 33342 signal was further optimized. Also for the channel 2 and 3, the background correction and values for the threshold of the GFP and mCherry signal were defined to exclude irregular and potentially false positive signals from the analysis. The translocation index (T_i_) was calculated as the ratio of the nuclear to the cytoplasmic signal intensity (T_i_ = CIRC∶RING) on a single cell basis (Figure 2A–C). As shown in Figure 2C/D, the T_i_ was highly significantly increased upon coexpression of Tasp- compared to inactive Tasp^T234V^-mCh. Notably, compared to analyzing only GFP/Hoechst 33342 double-positive cells the inclusion of GFP/mCherry/Hoechst 33342 triple-positive cells resulted in an improved T_i_. Based on the assay signal window and Z′-factor of 0.63, the criterion for the primary screen was set at a compound translocation index (T_ic_)>2 (T_ic_ = compound_(CIRC∶RING)_/DMSO_(CIRC∶RING)_). Only valid objects, i.e., cells that pass the object selection criteria (Figure 2A) were included in the analysis.

**Figure 3:**
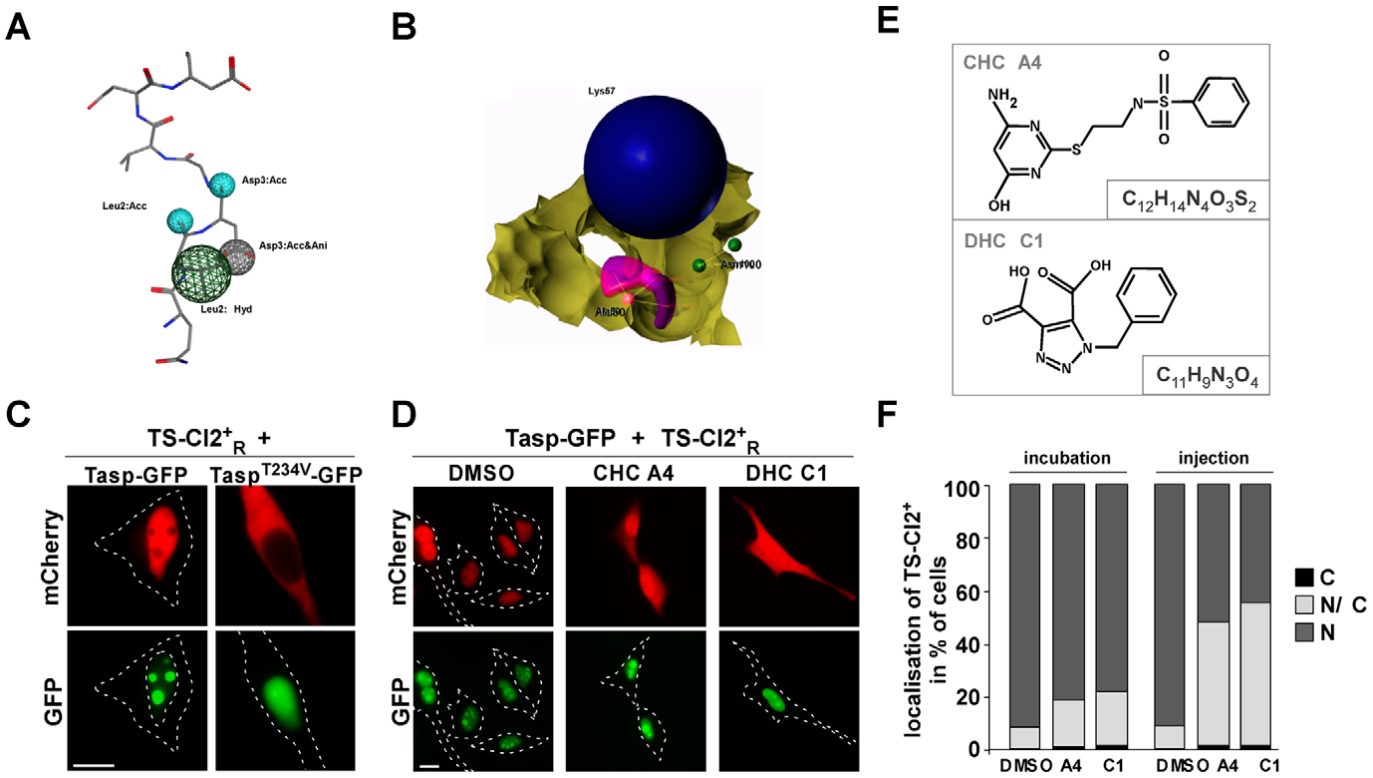
*In vivo* analysis of potential Taspase1 inhibitors. **A./B.** Pharmacophore-queries for virtual screening. **A.** Four-point MOE pharmacophore model based on the docked substrate QLDGVDD (shown as sticks coloured by element) together with the assigned pharmacophoric features (cyan: acceptor, green: hydrophobic, grey: acceptor and anion, left panel) **B.** Receptor-based SYBYL three-point pharmacophore model representing a negative charge (blue spheres) interacting with Lys^57^, hydrogen-bond donor (magenta) interacting with Ala^50^ and acceptor (green) interacting with Asn^100^ (right panel). The yellow surface indicates excluded volume. **C.** Expression of Tasp-GFP but not of inactive Tasp^T234V^-GFP induces nuclear accumulation of the red fluorescent biosensor, *TS-Cl2^+^_R_*, in HeLa transfectants. **D.** CHC-A4 and DHC-C1 partially inhibited *TS-Cl2^+^_R_* translocation. HeLa cell transfectants coexpressing *TS-Cl2^+^_R_* and Taspase1-GFP were treated with DMSO or compounds (50 µM final concentration), and analyzed 48 h later. Representative examples are shown. Scale bars, 10 µm. Dashed lines mark nuclear/cytoplasmic cell boundaries obtained from the corresponding phase contrast images. **E.** Structures and formulas of the respective compounds. **F.** Intracellular delivery of the compounds by microinjection resulted in enhanced inhibition. Vero cell transfectants coexpressing *TS-Cl2^+^_R_* and Taspase1-GFP were either treated with DMSO or compounds (50 µM final concentration) or microinjected. 48 h later, the percentage of cells showing cytoplasmic (C), cytoplasmic and nuclear (N/C) or nuclear (N) fluorescence was determined for at least 100 sensor expressing cells.

Next, we screened a focused compound library for potential Taspase1 inhibitors. As Taspase1 is not affected by general protease inhibitors [5], [17], we used a pharmacophor screening based on Taspase1's crystal structure and the model suggested by Khan *et al*. [6], [23]. In an attempt to preclude the binding of the peptidic substrate to Taspase1's active centre, two classes of low molecular weight (<500 Da) compounds were chosen (Suppl. Figure S3). The first group was named “deep hole compounds” (DHCs), as these substances were selected to hypothetically fit into the deep hydrophilic pocket of Taspase1 to prevent hydrolyzation of the substrate [6] (Figure 3A). The second class, “chloride hole compounds” (CHCs), should irreversibly occupy residues critical for chloride ion and substrate binding in Taspase1's active centre (Figure 3B). In addition, as historically the majority of new drugs have been derived from natural products [24] we also tested lipophilic extracts prepared from 90 different types of fungi obtained from the culture collection at the *IBWF* (http://www.ibwf.de) [25].

**Figure 4:**
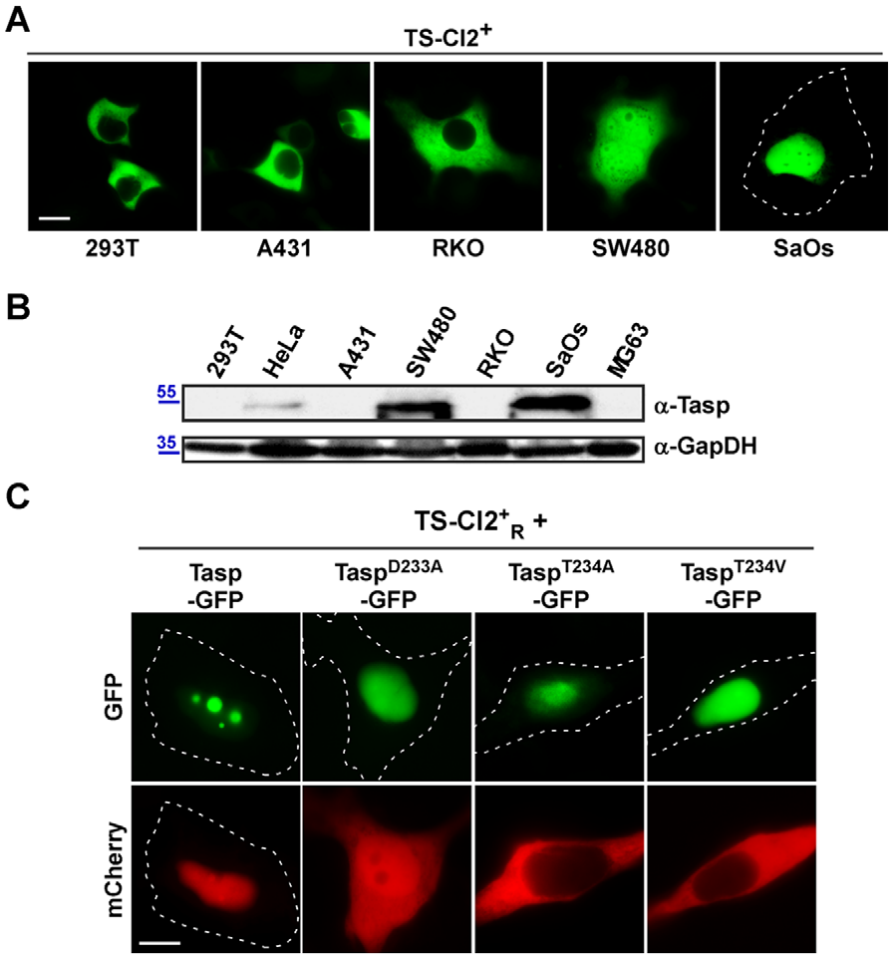
Biosensor-based probing of Taspase1 function *in vivo*. **A./B.** Cleavage of the biosensor correlated with endogenous Taspase1 levels in adherent tumor cell lines. **A.** Indicated cell lines were transfected with equal amounts of *TS-Cl2^+^* expression plasmid. 24 h later, localization of the biosensor was analyzed in at least 200 fluorescent cells displaying similar fluorescence intensity. Representative examples are shown. Cleavage-induced nuclear translocation differed significantly among tested cell lines. **B.** Endogenous Taspase1 levels were analysed by immunoblot using α-Tasp and -GAPDH Abs. **C.** Biosensor-based analysis of the proteolytic activity of Taspase1 variants in HeLa transfectants. Coexpression of Tasp^T234A^- or Tasp^T234V^-GFP fusion did not result in cleavage and nuclear accumulation of *TS-Cl2^+^_R_*. Tasp^D233A^-GFP displayed a reduced enzymatic activity compared to wt Taspase1-GFP. Scale bars, 10 µm. Dashed lines mark nuclear/cytoplasmic cell boundaries obtained from the corresponding phase contrast images.

Compounds and extracts were tested in 293T cells, which not express detectable levels of endogenous Taspase1 (Figure 4B). Cells coexpressing *TS-Cl2^+^* and Tasp-mCh or TS-Cl2^+^_R_ and Tasp-GFP (Figure 3C) were challenged in 96-well plates and analyzed 48 h after transfection to ensure that lack of inhibition is not due to slow intracellular entry rates of the substances. Although the majority of substances did not significantly affect Taspase1's *trans* cleavage activity at a concentration of 50 µM, we identified two compounds, N-[2-[(4-amino-6-oxo-3H-pyrimidin-2-yl)sulfanyl]ethyl]benzenesulfonamide (CHC-A4) and 2-benzyltriazole-4,5-dicarboxylic acid (DHC-C1), partially inhibiting biosensor translocation (Figure 3E). In contrast, the tested fungal extracts did not show detectable inhibition in our assay, although we observed cytotoxicity for some extracts (data not shown). As we previously identified transport inhibitors by chemicogenomic screens [20], we first verified that CHC-A4 and DHC-C1 did not affect nuclear import of the biosensor rather than cleavage. Treatment with LMB resulted in nuclear accumulation of *TS-Cl2^+^_R_* or *TS-Cl2^+^* even in the presence of the inhibitors, excluding interference with nuclear import (Suppl. Figure S2C and data not shown). Taspase1 inhibition could be confirmed in other cell lines using a compound concentration of 50 µM, although no inhibition was detectable at a concentration of 5 µM (Figure 3D and data not shown). Factors contributing to the weak inhibitory activity observed may be compound instability and/or their inefficient cell entry. Hence, to circumvent these limitations, we directly microinjected both compounds into *TS-Cl2^+^_R_*/Tasp-GFP expressing transfectants. Compared to adding the compounds directly to the cell culture medium, cytoplasmic injection of both compounds resulted in improved Taspase1 inhibition reducing nuclear translocation of the biosensor in the majority of cells (Figure 3F). The coinjected fluorescent Ab allowed to select only healthy cells for the analysis showing no signs of damage due to the microinjection procedure. In order to allow a comparison of both experimental approaches, the cells were inspected after 48 h. The reason why inhibition did not occur in all injected cells is not known, indicating that rational chemical modification of the primary hits is required to improve their activity.

### Biosensor-based probing of Taspase1 function

Besides their use in screening applications, we also exploited the biosensors as genetic tools to characterize Taspase1's biological functions.

First, we used the biosensor to probe expression and biological activity of endogenous Taspase1. As Taspase1 might also be relevant for solid tumors, we tested several cancer cell models. As depicted in Figure 4A/B, *TS-Cl2^+^* remained cytoplasmic in cell lines with low endogenous Taspase1 levels, whereas partial or complete nuclear translocation was evident in cell lines expressing high Taspase1 levels already after 24 h (for summary see Suppl. Table S2). Later time points did not show a different localization.

Second, we analyzed the proteolytic acivity of Taspase1 mutants, in which the catalytic nucleophile, Thr^234^, was changed into Val or Ala (Tasp^T234V^, Tasp^T234A^) or Asp^233^ was mutated into Ala (Tasp^D233A^). As shown in Figure 4C, coexpression of Tasp^T234V^- or Tasp^T234A^-GFP fusion did not result in cleavage and nuclear accumulation of *TS-Cl2^+^_R_* confirming that both mutants are catalytically inactive [5]. Notably, although *in vitro* studies reported a 1000-fold reduced activity for Tasp^D233A^ [5], the *in vivo* data indicated that Tasp^D233A^-GFP was still able to recognize and process the biosensor albeit with a somehow attenuated efficacy (Figure 4C).

**Figure 5:**
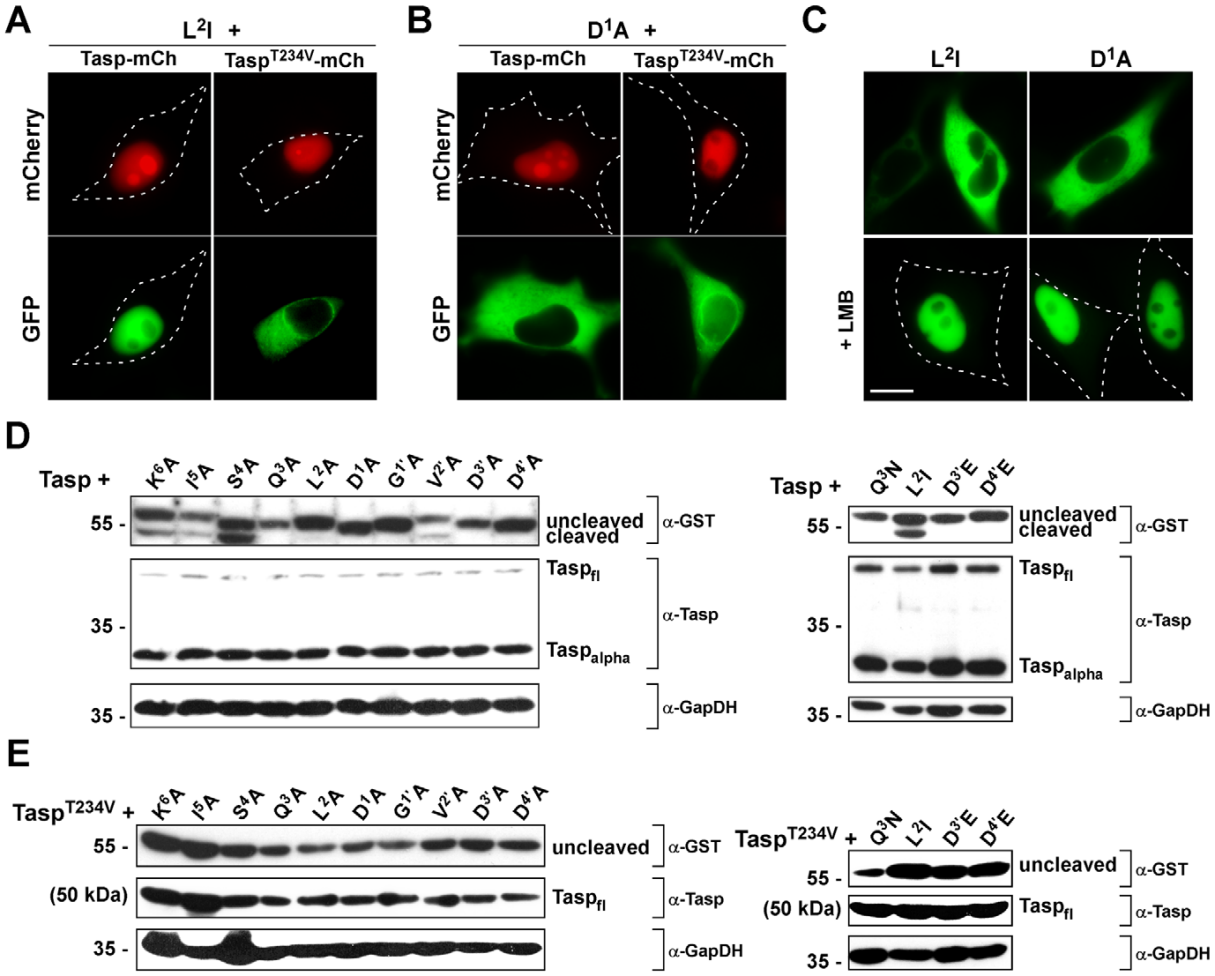
Identification of residues required for productive Taspase1 cleavage in living cells. **A.–C.** Nuclear translocation of the indicated biosensor cleavage site mutants (*TS-Cl2^+^_CSmut_*) was analyzed in HeLa transfectants coexpressing the indicated biosensors together with Tasp- or inactive Tasp^T234V^-mCherry. At least 200 fluorescent living cells were inspected, and representative examples are shown. Whereas substitution of Leu^2^ with Ile did not affect cleavage (**A.**), exchange of Asp^1^ with Ala completely abrogated cleavage (**B.**) LMB treatment verified that nuclear import of the variants was not affected. (**C.**) Scale bars, 10 µm. **D./E.** Cleavage of indicated cleavage site mutants by Tasp- (**D.**) or inactive Tasp^T234V^-mCh (**E.**) analyzed by immunoblot. Notably, D1′A, D3′A and D4′A mutants run lower in the gel, most likely due to the loss of the negative charge. Expression of Taspase1 proteins as well as of cleavage products in 293T cell lysates was visualized using α-GST or -Taspase1 Abs. GAPDH served as loading control.

Next, to uncover the sequence and spatial requirements for Taspase1 processing *in vivo*, we performed Ala scan mutagenesis of the MLL cleavage site (CS2; aa ^2713^KISQLD↓GVDD^2722^) in the biosensor background. As depicted in Figure 5, coexpression of the *TS-Cl2^+^* mutants (*TS-Cl2^+^_CSmut_*) with Tasp-mCh resulted in proteolytic cleavage and nuclear accumulation of only those biosensors in which non-essential residues were mutated. In contrast, changing critical aa into Ala almost completely prevented cleavage and nuclear accumulation of the autofluorescent proteins, leading to the following consensus sequence: K**^6^**I**^5^**S**^4^Q^3^L^2^D^1^↓G^1′^**V^2′^ **D^3′^D^4′^** (essential aa in bold; see Table 1 for summarized results of targets). Notably, even replacing critical residues by chemically similar aa could not rescue cleavage, exempt the exchange of Leu for the also hydrophobic aa Ile or Val (Figure 5A/B and Table 1). These results could be confirmed by immunoblot analysis (Figure 5D/E). Again, specificity of the assay was verified by cotransfection of inactive Tasp^T234V^-mCh, which did neither result in cleavage nor nuclear accumulation of the biosensors (Suppl. Figure S2C). Nuclear accumulation of all the *TS-Cl2^+^_CSmut_* variants upon LMB treatment further excluded the formal possibility that mutagenesis had affected import of the biosensors (Figure 5C). Collectively, these results underline the reliability and practical advantages of our visual cell based assay to probe Taspase1 function in living cells.

**Table 1:** Cleavage-site residues critical for Taspase1 processing *in vivo*.

Cleavage-site mutation	*In cell* cleavage by Taspase1
K^6^A	+
I^5^A	+
**S^4^A**	**+**
**Q^3^A**	**−**
**Q^3^N**	**−**
**L^2^A**	**−**
L^2^I	+
**D^1^A**	**−**
**G^1′^A**	**−**
V^2′^A	+
**D^3′^A**	**(−)**
**D^3′^E**	**(−)**
**D^4′^A**	**(−)**
**D^4′^E**	**(−)**

Summary of results obtained from the biosensor-based mapping.

Cleavage site aa residues: K**^6^**I**^5^**S**^4^Q^3^L^2^D^1^↓G^1′^**V^2′^ **D^3′^D^4′^** (essential aa in bold). −: no cleavage, (−): reduced cleavage, +: cleavage.

### Identification of novel human Taspase1 targets

To bioinformatically identify novel human Taspase1 targets, we used the motifs Q^3^[I,L,V]^2^D^1^↓G^1′^X^2′^X^3′^D^4′^ and Q^3^[I,L,V]^2^D^1^↓G^1′^X^2′^D^3′^X^4′^ obtained by our mutational analysis to scan the *Swiss-Prot* database. Besides the expected Taspase1 targets, MLL1 and MLL4, our analysis identified TF2A, the FERM Domain-Containing Protein 4B (FRM4B), the Tyrosine-Protein Phosphatase Zeta (PTRZ) and DNA Polymerase Zeta (DPOLZ) as putative Taspase1 substrates (see Table 2 for verified, Suppl. Table S3 for predicted targets).

**Table 2:** Characteristics of verified Taspase1 target genes according to their GO term classifications.

Gene Locus	GO : biological process		GO : cellular component		GO : molecular function	
	ID	description	ID	description	ID	description
MLL1	0006366	transcription	0071339	nucleoplasm	0003680	DNA binding
	0006461	protein complex assembly			0003700	nucleic acid binding
	0006915	cell death			0003702	transcription regulator activity
	0035162	developmental process			0008270	ion binding
	0043984	histone acetylation			0042800	N-methyltransferase activity
	0051568	histone methylation			0042803	protein homodimerization activity
					0045322	transcription factor activity
					0070577	histone binding
MLL4	0006350	transcription	0035097	nucleoplasm	0005515	protein binding
	0016568	chromatin modification			0008270	ion binding
	0008284	cell proliferation			0010843	DNA binding
	0033148	estrogen signalling			0018024	N-methyltransferase activity
	0010552	transcription				
	0043627	estrogen signalling				
FRM4B	**-**	unknown	0005737	cytoplasm	0005488	binding
			0005856	cytoskeleton		
PTRZ	0006470	metabolic process	0005887	integral to membrane	0005001	phosphatase activity
	0007417	developmental process			0005515	protein binding
					0008330	phosphatase activity
TF2AA	0006367	transcription initiation	0005634	nucleus	0003677	DNA binding
	0006368	RNA elongation	0005672	nucleoplasm	0003713	transcription regulator activity
	0032568	transcription	0005737	cytoplasm	0016251	transcription regulator activity
	0045449	transcription			0017025	TATA-binding protein binding
					0046982	protein heterodimerization activity
DPOLZ	0006261	DNA replication	0005634	nucleus	0000166	nucleotide binding
	0006281	DNA repair			0003677	DNA polymerase activity
					0003887	DNA polymerase activity
					0008270	ion binding

**Figure 6:**
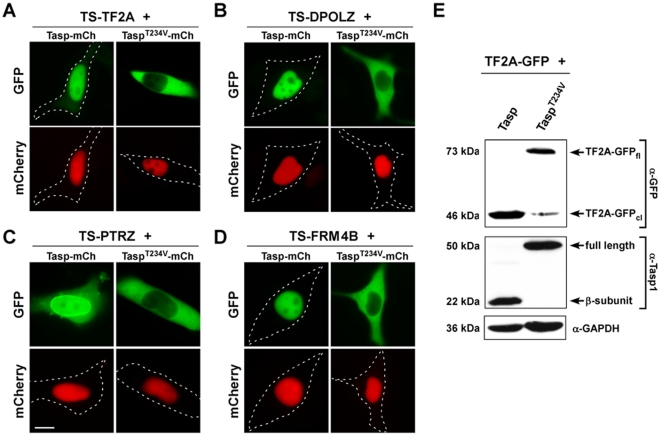
Analysis of Taspase1-mediated cleavage of *in silico* predicted targets. **A.** HeLa cells were transfected with biosensors harboring full length TFIIA (*TS-TF2A*), the predicted cleavage site from DPOLZ (*TS-DPOLZ*), PTRZ (*TS-*PTRZ), or FRM4B (*TS-*FRM4B) together with the Tasp-mCh or inactive Tasp^T234V^-mCh expression plasmid. 24 h later, *trans*-cleavage resulting in nuclear translocation of the biosensors was analyzed in at least 200 fluorescent living cells. Representative examples are shown. Productive cleavage was confirmed for all targets. Scale bars, 10 µm. **B.** Processing of full length TFIIA-GFP upon coexpression of Taspase1 but not of the inactive Tasp^T234V^ mutant shown by immunoblot analysis of transfected 293T cell lysates. Protein expression and cleavage was visualized by α-GFP and α-Taspase1 Ab. GAPDH served as loading control.

To experimentally verify that these proteins represent most likely biologically relevant novel Taspase1 substrates, we first tested the cleavage sites of these targets in the biosensor system. Indeed, integration of the cleavage sites from FRM4B, PTRZ as well as DPOLZ resulted in cytoplasmic biosensors, which were efficiently recognized and processed by Taspase1 (Figure 6A–6D left panels) under the same experimental conditions as the developed TS-Cl2^+^ biosensor. Coexpression of the inactive Tasp^T234V^ mutant confirmed the specificity of the assay (Figure 6A–6D right panels). Immunoblot analysis further demonstrated that also full length TFIIA-GFP was efficiently processed upon coexpression of Taspase1, underlining the *in vivo* relevance of the *in silico* prediction (Figure 6E).

## Discussion

A critical requirement to understand the biological processes a protease participates in is to dissect the mechanisms of protease activity, as well as the biochemistry that relates their structure to function [2], [26]. Various strategies including genetics, proteomics and *in silico* biology are currently pursued to achieve these goals [1], [27]. Although Taspase1 was identified as the protease responsible for the cleavage of the MLL protein [5], [8], [10], relatively little is still known about its (patho)biological relevance. This is in contrast to other disease relevant proteases, such as matrix metalloproteinases, which were the first protease targets considered for combating cancer because of their role in extracellular matrix degradation [1], [28]. Besides the complexity of (patho)biological processes Taspase1 might be involved in ([5], [8], [10], this study), our knowledge is currently limited by the fact that neither efficient Taspase1 inhibitors nor assay systems applicable for the high-throughput identification of such chemical decoys are available. In order to successfully employ chemogenomics, cell based assays appear to be particularly relevant for investigating Taspase1. Previous *in vitro* cleavage assays were rather inefficient or operated with purified or *in vitro* translated enzyme, and thus are not amenable for high-throughput applications (this study, [6], [17]). The reasons for the observed improved performance of the *in vivo* biosensor assay in this study may be multifold, including the possibility that Taspase1 produced in bacteria shows reduced catalytic activity due to partial denaturation. Also, a chloride ion, described to be interacting with the amino acids Gly^49^, Gln^100^ and Thr^234^ of recombinant Taspase1 [6] may act as a competitive inhibitor under *in vitro* assay conditions. Although we are currently lacking experimental evidence it is suffice to speculate that eukaryotic post-translational modifications and/or co-factors may be required to render the enzyme fully active. Nevertheless, our results underlined the practical advantages and biological relevance of the cellular assay to investigate Taspase1 function.

A key part of understanding protease signaling in both health and disease is to identify a protease's physiological substrates. Although the sequence Q^3^X^2^D^1^↓G^1′^ has been proposed as a consensus cleavage site sequence for Taspase1 [6], employing this motif for the bioinformatic identification of novel Taspase1 targets is impractical, as more than 1000 putative substrates were predicted. To improve our understanding of Taspase1's substrate specificity, we used our biosensor assay combined with positional scanning mutagenesis to identify residues essential for Taspase1 cleavage activity in living cells. As expected, Asp at the P^1^ position was required for cleavage by this aspartase, and Gly at P1′ did not even tolerate its replacement by Ala. Also, Gln at position P^3^ was critical for substrate recognition, as an exchange of this uncharged polar amino acid by the smaller hydrophobic residue Ala or even the similar but smaller amino acid Asn completely blocks cleavage. In contrast to previous studies [6], we found that albeit position P^2^ can hold hydrophobic residues of similar size (Leu, Ile, Val), other amino acids such as the smaller hydrophobic amino acid Ala were not tolerated. Hence, hydrophobicity in combination with certain size are likely to be structural requirements for productive cleavage. Position P^2′^ was found to be flexible, whereas the amino acids at P^3′^ and P^4′^ seem to be interdependent. At least one of these residues needed to be Asp, although a small residue at the other position, like Gly or Ala, was tolerated. Glu at either position however impaired cleavage, indicating that not only charge but also size is important for productive processing. Taken together, we defined the sequence motif Q^3^[I,L,V]^2^D^1^↓G^1′^V^2′^D^3′^D^4′^ as an improved consensus recognition site for Taspase1.

Employing this motif, we bioinformatically identified not only known Taspase1 substrates, such as MLL1 and MLL4, but also proteins, which have not been considered as potential targets for this protease. These include the FERM Domain-Containing Protein 4B (FRM4B), the Tyrosine-Protein Phosphatase Zeta (PTRZ) and DNA Polymerase Zeta (DPOLZ), suggested to be relevant for various biological processes (Table 2). Although we are currently lacking experimental evidence how Taspase1-mediated processing of these targets contributes to their functional regulation, we could confirm that the cleavage sites of these proteins are recognized and processed by Taspase1 *in vivo*. The potential impact of Taspase1 for neoplastic diseases extrapolated from its processing of leukemia inducing MLL fusion proteins containing a functional Taspase1 cleavage site is further supported by our identification of these substrates. We just showed that only AF4•MLL but not the reciprocal translocation product, MLL•AF4, lacking the Taspase1 cleavage site, can cause proB ALL in a murine model [13]. Albeit the exact biological relevance of PTRZ for disease and development is not yet resolved, this phosphatase was suggested as a therapeutic target for glioblastoma and glioblastoma-derived stem cells [29], [30]. Likewise, although the function of FRM4B is unknown, other members of the protein 4.1 superfamily such as FRMD4A or FRMD3 have been implicated in oncogenic signaling [31], [32], [33]. Notably, DPOLZ is not only essential during embryogenesis but also important in defense against genotoxins. As recent evidence indicates that reduced DPOLZ levels enhance spontaneous tumorigenesis, it is tempting to speculate that Taspase1 might participate in controlling DPOLZ levels and thus, disease [34], [35]. Notably, we found that Taspase1 is expressed in several solid tumor cell models. Whether the differences in Taspase1 expression levels detected have implications also on the (patho)biological characteristics of the tumor cell lines as well as for the primary disease remains to be investigated.

Nevertheless, there is increasing evidence that Taspase1 may be critically contributing to disease, underlining its pathobiological and potentially therapeutic relevance. However, we still do not comprehense the processes and molecular mechanisms Taspase1 might be involved in. Thus, besides genetic and biochemical approaches, small molecules allowing a (transient) chemical knockout of Taspase1 in a specific biological system or disease model would be highly valuable. These needs underline the relevance of the developed translocation biosensor for the identification and validation of inhibitors in living cells. Importantly, the biosensors can operate with red or green autofluorescent proteins, which can be optimally detected even by high-throughput fluorescence microscopy, and are not restricted to a specific cell type. The assay strictly depends on the presence of catalytically active Taspase1 and occurs with a high signal-to-noise ratio, allowing its use in HTS/HCS applications of large or focused compound libraries.

As a proof of principle, we screened a collection of small molecules, which were chosen based on a pharmacophore screening relying on the published crystal structure of Taspase1 [6]. The low molecular weight compounds were selected by virtual screening to prevent substrate cleavage and/or arrest the enzyme in an inactive state. Noteworthy, we identified two substances showing inhibitory activity in living cells, which would represent a primary hit rate of 3%. The reasons why other compounds were not active in our assay are versatile, including their potential inability to penetrate cell membranes. Also, the accuracy of virtual screening might have been flawed as details in the published crystal structure of Taspase1 are missing and the catalytic mechanism of Taspase1 is not yet resolved in detail. The first hit compound, N-[2-[(4-amino-6-oxo-3H-pyrimidin-2-yl)sulfanyl]ethyl]benzenesulfonamide (CHC-A4), was retrieved by SYBYL UNITY-Flex similarity searching (receptor-derived pharmacophore model). The second, 2-benzyltriazole-4,5-dicarboxylic acid (DHC-C1), was selected based on the four-point substrate pharmacophore model using the software Molecular Operating Environment. Both compounds are small and polar, with a pronounced hydrogen-bonding potential, which can be readily explained by the requirements of the pharmacophore queries. Although we controlled that the compounds do not unspecifically act by blocking nuclear import of the biosensors, significant Taspase1 inhibition *in vivo* required relative high inhibitor concentrations (50 µM). Notably, we observed improved inhibition upon direct delivery of both compounds into the cells by microinjection, indicating that the weak inhibitory activity observed may be due to compound instability and/or their inefficient cell entry. Recently, Lee *et* al. [17] designed chemically modified peptidic derivates of a Taspase1 cleavage substrate. Although some of these compounds displayed mild inhibitory activity using *in vitro* Taspase1 assays (e.g., yzm18 IC_50_29.4 µM), these peptide-based inhibitors have not shown efficacy in living cells, in contrast to our low molecular weight inhibitors.

Although natural products appear to interrogate a different area of chemical space than synthetic compounds [36], the tested lipophilic fungal extracts showed no inhibitory activity. Failure may be due to the fact that albeit such extracts contain a mixture of many different substances, the concentration of potentially active ingredients may be too low or outweighed by toxic effects of other components. Also, the numbers of samples which have to be screened in unfocussed natural product libraries are usually high, and hit rates are mostly below 0.01% [20], [24].

Hence, as future strategies to identify potent Taspase1 inhibitors we suggest to focus on a rational synthesis of derivates based on the structures of our primary hits combined with HTS of large natural/synthetic compound libraries.

## Materials and Methods

### Antibodies (Ab), reagents, compounds and fungal extracts

Ab used: α-GST (sc-57753), α-Taspase1 (sc-85945), α-GAPDH (sc-47724) and α-GFP (sc-8334) (Santa Cruz Biotechnology, Heidelberg, Germany); α-myc-tag (NEB GmbH, Frankfurt am Main, Germany). Appropriate HRP-, Cy3- or AlexaDye-conjugated secondary antibodies (Sigma Aldrich, Munich, Germany; Santa Cruz Biotechnology, Heidelberg, Germany) were used. Reagents were from Sigma Aldrich (Sigma Aldrich, Munich, Germany) unless stated otherwise. Cells were treated with leptomycin B (LMB) (10 nM) as described in [37]. Potential Taspase1 inhibitors (Suppl. Figure S3) were purchased from ASINEX Ltd (Moscow, Russia). Fungal extracts were obtained from submerged cultures of higher fungi, preferentially from asco- and basidiomycetes, deposited in the culture collection at the *IBWF*, as described [25]. Briefly, the fermentation of the fungi was stopped as soon as free glucose in the growth medium was depleted, and mycelia were separated by filtration. Lipophilic molecules were extracted from the culture broth with ethyl acetate. The extracts were dried *in vacuo*, redissolved in 25 µL DMSO, and aliquots of these extracts were used in the assays at a dilution of 1∶2000.

### Cell culture, microscopy and fluorescence imaging of cells

Cell lines used in the study were maintained and transfected as described [20], [37]. MEF3T3 stably expressing the Dox-inducible *TS-Cl2^+^_TRE_* were established by G418- (800 µg/mL) and puromycin- (2 µg/mL) selection, and fluorescence activated cell sorting as reported [20]. Cells were cultured in medium containing 1 µg/mL doxycycline (Dox) [20]. Twelve-bit black and white images were captured using a digital Axiocam CCD camera (Carl Zeiss, Jena, Germany). Quantitation, image analysis and presentation were performed as described [18], [38]. The nuclear signal was similarly obtained by measuring the pixel intensity in the nucleus. Nuclei were marked by Hoechst 33258 staining as described [18], [39]. To determine the average intracellular protein localization, at least 200 fluorescent cells from three separate images were examined in three independent experiments. The number of cells exhibiting cytoplasmic (C, cytoplasmic signal >75% of the total cellular signal), cytoplasmic and nuclear (C/N), or nuclear (N, nuclear signal >75% of the total cellular signal) fluorescence was counted.

### Computer-assisted microinjection

Vero cells were transfected with TS-Cl2^+^_R_ and Taspase1-GFP expression plasmids (1 µg each). 4 h after transfection, DMSO or compounds (200 µM concentration in DMSO) were microinjected into the cytoplasm as described in detail [40]. An Alexa350-labelled α-IgG Ab (0.5 µg/µL) served to mark injected cells. 48 h later, the percentage of cells showing cytoplasmic (C), cytoplasmic and nuclear (N/C) or nuclear (N) fluorescence was determined for at least 100 GFP/mCherry-positive and injected cells.

### Cellomics ArrayScan® VTI-based HCS

Automated analysis of the molecular translocation assay was performed using the Cellomics ArrayScan® VTI Imaging Platform (Thermo Fisher Scientific Inc., Berkshire, UK). Cells were seeded with an electronic multichannel pipette (Eppendorf, Hamburg, Germany) into black-walled 96-well thin bottom Greiner μclear® plates (Greiner, Frickenhausen, Germany) and incubated at 37°C, 5% CO_2_ and 95% humidity. Cells were transfected, and compounds (50 µM final concentration dissolved in DMSO) were added 4 h later. For each experiment two wells were drug treated, and each experiment was performed in duplicates. DMSO was used as a control. 48 h later, cells were fixed by the addition of 50 µL 4% PFA, and nuclei were stained by addition of Hoechst 33342 at a final concentration of 40 µM for 10 min. After a final wash with PBS, 50 µL PBS were left in each well and the plates were sealed and stored at 4°C. Images were acquired and analyzed on the Cellomics ArrayScan® VTI Imaging Platform as described [20]. Briefly, binary image masks were created for GFP, mCherry and Hoechst 33342 positive staining to define regions of interest (ROI) for analysis. For this purpose, we applyed a median filter (3×3 pixel radius) to remove noise and to approximate the distribution of staining intensity to a median value. Automatic thresholding using the Isodata algorithm was used to convert the image to a binary mask that included all fluorescence data above background [20]. The Hoechst 33342 staining (channel 1) mask was used to define the nuclear ROI. Subsequently, the Hoechst 33342 mask was subtracted from the GFP mask (channel 2) to create a staining mask defining the cytoplasmic ROI. Scans were performed sequentially with settings to give sub-saturating fluorescence intensity, and a minimum of 400 valid objects per well was recorded.

### Plasmids

To generate plasmid p_NLS-GFP/GST-CS2-NES_Rev_ (p_*TS-Cl2^+^*), encoding a fusion composed of the SV40 large T-antigen NLS, GST, GFP, the Taspase1 cleavage site from MLL (CS2; aa ^2713^KISQLDGVDD^2722^), and a Myc-epitope-tagged NES from the HIV-1 Rev protein (NES_Rev_) [41], the CS2 coding sequence was inserted into vector pNLS-GFP/GST-CS3-RevNES (p_BS-Casp3), replacing CS3. p_BS-Casp3 encodes a biosensor harboring the cleavage site for Caspase3 (CS3: aa KRKGDEVDGVDE) [18]. p_*TS-Cl2^+^_R_* encodes a red fluorescent biosensor (NLS-mCherry/GST-CS2-NES_Rev_), in which GFP was replaced by mCherry [18]. Expression plasmids for *TS-Cl2^+^* variants, in which CS2 was mutated (p_*TS-Cl2^+^_mut_*; see Table 2, and Suppl. Table S1 for oligonucleotides used) were generated by oligonucleotide-annealing and cloning into the *NotI/XhoI*-restriction sites of p_*TS-Cl2^+^* as described [18]. Likewise, the coding sequence for full length TFIIA (p_*TS-TF2A*), or the cleavage sites from DPOLZ (p_*TS-DPOLZ*), PTRZ (p_*TS-PTRZ*) or FRM4B (p_*TS-FRM4B*) were inserted into p_TS-Cl2^+^, thereby replacing the CS2. pTRE-NLS-GFP/GST-CS2-NES_Rev_ (p_*TS-Cl2^+^_TRE_*) allows the inducible expression of the biosensor (“tet-off”) and was constructed by inserting the NLS-GFP/GST-CS2-NES_Rev_ coding sequence into pTRE-NLS-GFP/GST-NES_Rev_ [18].

The Taspase1 or TFIIA coding sequence was amplified from cDNA obtained from a human head and neck tumor. mRNA preparation and cDNA synthesis from tumor tissue was performed as described [42]. Cloning of the Taspase1 coding sequence into expression vectors pc3, pc3-GFP, pc3-BFP, and pc3-mCherry using *BamHI/EcoRI*- or *BamHI/NheI-*restriction sites, respectively, allowed the expression of Taspase1, alone or as a fusion with fluorescent proteins as described [21], [43]. Plasmid p_Tasp^T234V^-GFP, p_Tasp^T234A^-GFP, p_Tasp^D233A^-GFP and p_Tasp^T234V^-mCherry or –BFP encoding the catalytically inactive Taspase1 mutant, were generated by splice overlap extension polymerase chain reaction as reported [18], [44]. p_TFIIA-GFP, encoding a TFIIA-GFP fusion, was generated by PCR amplification and cloning into pc3-GFP as reported [45]. pc3_RevM10BL-BFP, encoding a mutant HIV-1 Rev protein, was described [40].

Bacterial expression plasmid pGEX_GST-Tasp1-GFP encoding a GST-Tasp1-GFP fusion protein and pET22-Tasp, encoding a His-tagged Taspase1 protein, were generated by inserting the Taspase1 coding sequence into pGEX-GFP or pET22b+, respectively, as reported [46]. The coding sequence for the MLL aa 2650–2808 (2Cl) was inserted into vector pGEX5T to generate pGEX5T_GST-2Cl, encoding a His-tagged-GST-2Cl fusion protein.

Plasmids were verified by sequence analysis as described [47]. Oligonucleotides used for PCR amplification and cloning are listed in Suppl. Table S1.

### Protein extraction, immunoblot analysis and immunofluorescence

Preparation of whole cell lysates and immunoblotting were carried out as described [39], [48]. Immunofluorescence was performed as reported in detail [18], [49].

### *In vitro* Taspase1 cleavage assay

His-tagged Taspase1, GST-Taspase1-GFP and His-tagged GST-2Cl substrates were expressed in BL21 bacteria and purified by nickel chelating or glutathione affinity chromatography as described [18], [46]. Fractions were eluted (50 mM NaH_2_PO_4_, 300 mM NaCl, 250 mM Imidazol, pH 8.0) and dialyzed against Taspase1 cleavage buffer (200 mM Hepes/KOH pH 7.9, 10 mM MgCl_2_, 40 mM KCl, 20% Saccharose, 10 mM DTT). Trans-cleavage assays were performed in cleavage buffer adding recombinant Taspase1 to 5 µM of GST-2Cl. Analysis of cleavage was carried out by SDS page followed by Coomassie staining as outlined in [49].

### Virtual screening and database searches

An X-ray structure of the inactive autocatalytically processed Taspase 1 dimer (Protein Data Bank ID 2a8j, 1.9 Å resolution [6] served as the basis for pharmacophore model generation and computer-based similarity searching in a commercial screening compound collection (Asinex Gold collection nov. 2005: 231,812 compounds; ASINEX Ltd, Moscow, Russia) [23]. Briefly, screening compounds were reduced to “druglike” compounds (clog*S*>4, no rule-of-five violation) using *Molecular Operating Environment* (MOE) 2005.06 (Chemical Computing Group, Montreal, Canada), and for the remaining 181,403 substances single conformers were computed using CORINA 3.20 (Molecular Networks GmbH, Erlangen, Germany). Bases were de-protonated, acid groups were protonated (“wash” function in MOE). Two types of pharmacophore hypotheses were generated: (i) “ligand-based” models from hypothetical binding mode of the Taspase1 cleavage site substrate QLD↓GVDD [5], (pre-docking of the substrate by GOLD 3.0.1; force-field relaxation using AMBER99 in MOE; manual assignment of potential pharmacophoric points in MOE; similarity searching with MOE), and (ii) a “receptor-based” model of a hypothetical ligand pharmacophore using SYBYL 7.1 (Tripos Inc, Missouri, U.S.A.), with UNITY-Flex search option. The resulting ligand-based pharmacophore models yielded a total of 62 perfect matches in the screening compound collection, and the receptor-based model retrieved 209 perfect matches. From these hits, compounds were selected for testing.

For the bioinformatic identification of potential human Taspase1 targets, *ScanProsite* searches were performed in the *human* taxon of the *UniProtKB/SwissProt* database using the patterns Q-[IVL]-D-G-X-D-D, Q-[IVL]-D-G-X-X-D and Q-[IVL]-D-G-X-D-X as queries.

### Statistical analysis

For experiments stating *p*-values, a paired Student's t-test was performed. Unless stated otherwise, *p*-values represent data obtained from three independent experiments done in triplicate. *p-*values<0.05 were considered as significant.

## Supporting Information

Figure S1 ***In vitro*** **Taspase1 cleavage assay.** **A.** Extensive aggregation of GST-Tasp1-GFP expressed in BL21 bacteria visualized by fluorescence microscopy. In contrast, GST-GFP showed no aggregation and was efficiently expressed. Images were taken with identical CCD camera settings. Scale bars, 1 µm. **B.** Schematic representation of expression constructs for His-tagged Taspase1 (rTasp) and the substrate GST-2Cl, containing the MLL cleavage sites CS1 and CS2 (MLL aa 2650–2808). Molecular weight of the expected cleavage products is indicated. **C.** Concentration dependent processing of GST-2Cl by recombinant Taspase1 (rTasp). GST-2Cl (5 µM) was incubated with increasing amounts of rTasp (lane1: 2.5 µM, 2: 1.25 µM, 3: 0.63 µM, 4: 0.32 µM, 5: 0.16 µM, 6: 0.08 µM, 7: 0.04 µM, 8: 0.02 µM) for 60 min. Cleavage was visualized by SDS-PAGE and Coomassie staining. Uncleaved and cleaved proteins are indicated. **D.** Time dependent processing of GST-2Cl by recombinant Taspase1. GST-2Cl (5 µM) was incubated with 2.5 µM Taspase1, and cleavage was monitored over time. Cleavage was visualized by SDS-PAGE and Coomassie staining. Uncleaved and cleaved proteins are indicated.
(CVX)

Figure S2 ***In vivo*** **screening for inhibitors of Taspase1 activity.** **A.** Principle of the inducible biosensor system, Tet-off *TS-Cl2^+^_TRE_*. Dox interacts with tTA preventing its binding and thus activation of the TRE-containing CMV promoter. Removal of Dox allows tTA binding, triggering transcriptional activation and expression of the shuttling biosensor, which predominately localizes to the cytoplasm. Dox, Doxycylin; pCMV/pCMV_min_, (minimal) CMV promoter; TRE, Tetracycline-responsive promoter element; tTA, Tet-controlled transactivator. **B.** Dox-induced biosensor expression. MEF3T3 cells stably expressing *TS-Cl2^+^_TRE_* were cultured in the presence or absence of Dox. In presence of Dox, no expression of the biosensor is detectable. Three days after Dox removal, expression of cytoplasmic *TS-Cl2^+^_TRE_* is visible (−Dox 3d), and cleavage by endogenous Taspase1 results in its nuclear accumulation 24 h later (−Dox 4d). Living cells were analyzed by fluorescence microscopy and images taken with identical CCD camera settings. Scale bars, 10 µm. **C.** CHC-A4 or DHC-C1 do not interfere with nuclear import of the biosensor. HeLa transfectants were treated with DMSO or compounds (50 µM final concentration) for 12 h. Treatment with the export inhibitor LMB (10 nM, 2 h) resulted in efficient nuclear accumulation of *TS-Cl2+_R_* even in the presence of the compounds. Localization of *TS-Cl2+_R_* was analyzed in at least 200 fluorescent cells. Representative examples are shown. Scale bars, 10 µm.
(TIF)

Figure S3 **Chloride and deep hole compounds analyzed in HCS assay.** Chemical structures and formulas are shown. Abbreviations: CHC, chloride hole compound; DHC, deep hole compound.
(TIF)

Table S1 **Oligonucleotides used for PCR amplification and cloning.**
(DOC)

Table S2 **Taspase1 expression levels and proteolytic activity in solid cancer cell line models.**
(DOC)

Table S3 **List of potential human Taspase1 targets predicted by** ***ScanProsite*** **.**
(DOC)
