# Supplementary figures and images for: Bioassays to Monitor Taspase1 Function for the Identification of Pharmacogenetic Inhibitors
Source: PLoS One. 2011 May 25;6(5):e18253. doi: 10.1371/journal.pone.0018253 (PMC3102056; doi:10.1371/journal.pone.0018253)

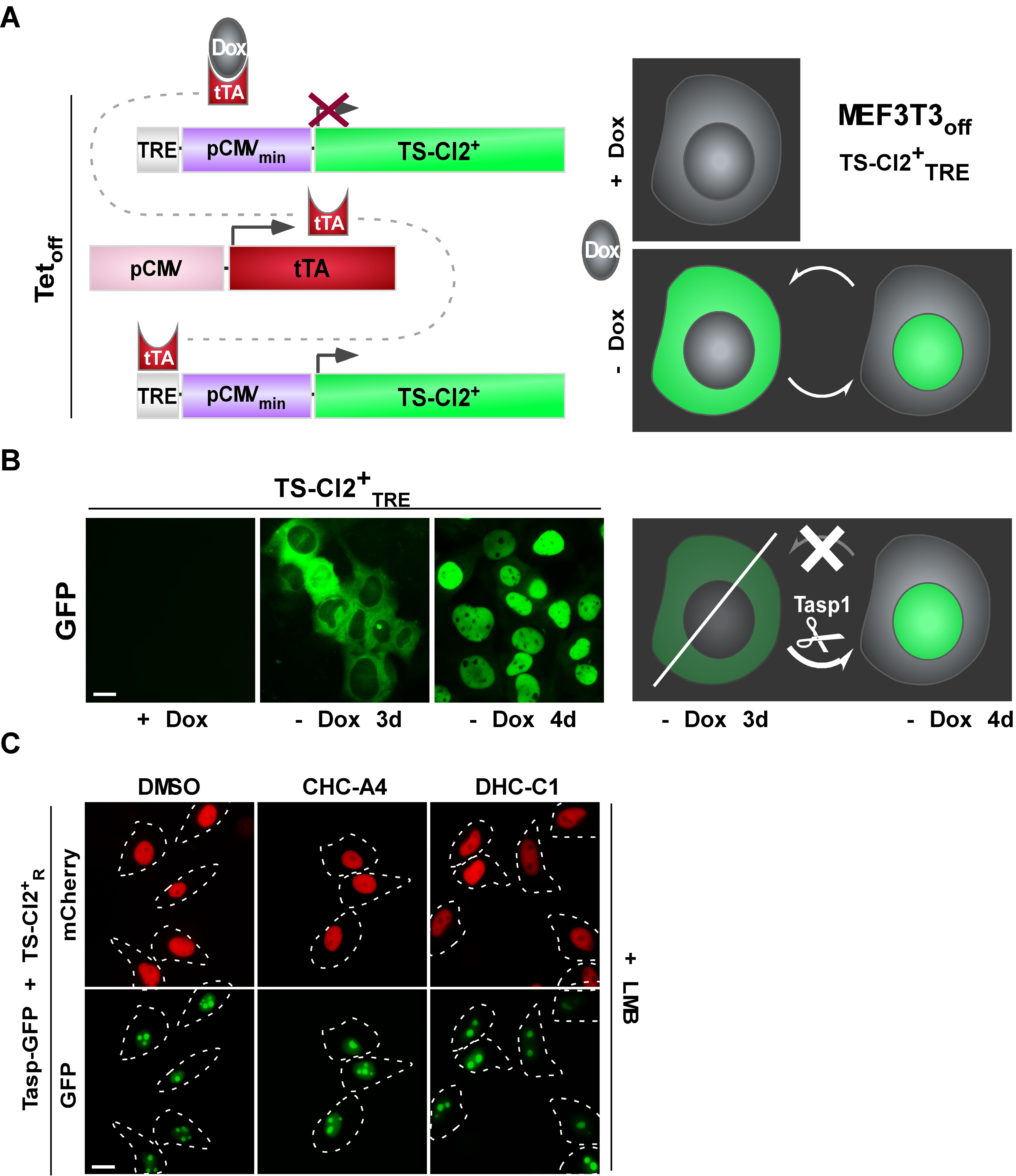

Supplement: Figure S2 — In vivo screening for inhibitors of Taspase1 activity. A. Principle of the inducible biosensor system, Tet-off TS-Cl2+TRE. Dox interacts with tTA preventing its binding and thus activation of the TRE-containing CMV promoter. Removal of Dox allows tTA binding, triggering transcriptional activation and expression of the shuttling biosensor, which predominately localizes to the cytoplasm. Dox, Doxycylin; pCMV/pCMVmin, (minimal) CMV promoter; TRE, Tetracycline-responsive promoter element; tTA, Tet-controlled transactivator. B. Dox-induced biosensor expression. MEF3T3 cells stably expressing TS-Cl2+TRE were cultured in the presence or absence of Dox. In presence of Dox, no expression of the biosensor is detectable. Three days after Dox removal, expression of cytoplasmic TS-Cl2+TRE is visible (−Dox 3d), and cleavage by endogenous Taspase1 results in its nuclear accumulation 24 h later (−Dox 4d). Living cells were analyzed by fluorescence microscopy and images taken with identical CCD camera settings. Scale bars, 10 µm. C. CHC-A4 or DHC-C1 do not interfere with nuclear import of the biosensor. HeLa transfectants were treated with DMSO or compounds (50 µM final concentration) for 12 h. Treatment with the export inhibitor LMB (10 nM, 2 h) resulted in efficient nuclear accumulation of TS-Cl2+R even in the presence of the compounds. Localization of TS-Cl2+R was analyzed in at least 200 fluorescent cells. Representative examples are shown. Scale bars, 10 µm. (TIF) [file pone.0018253.s002.tif]

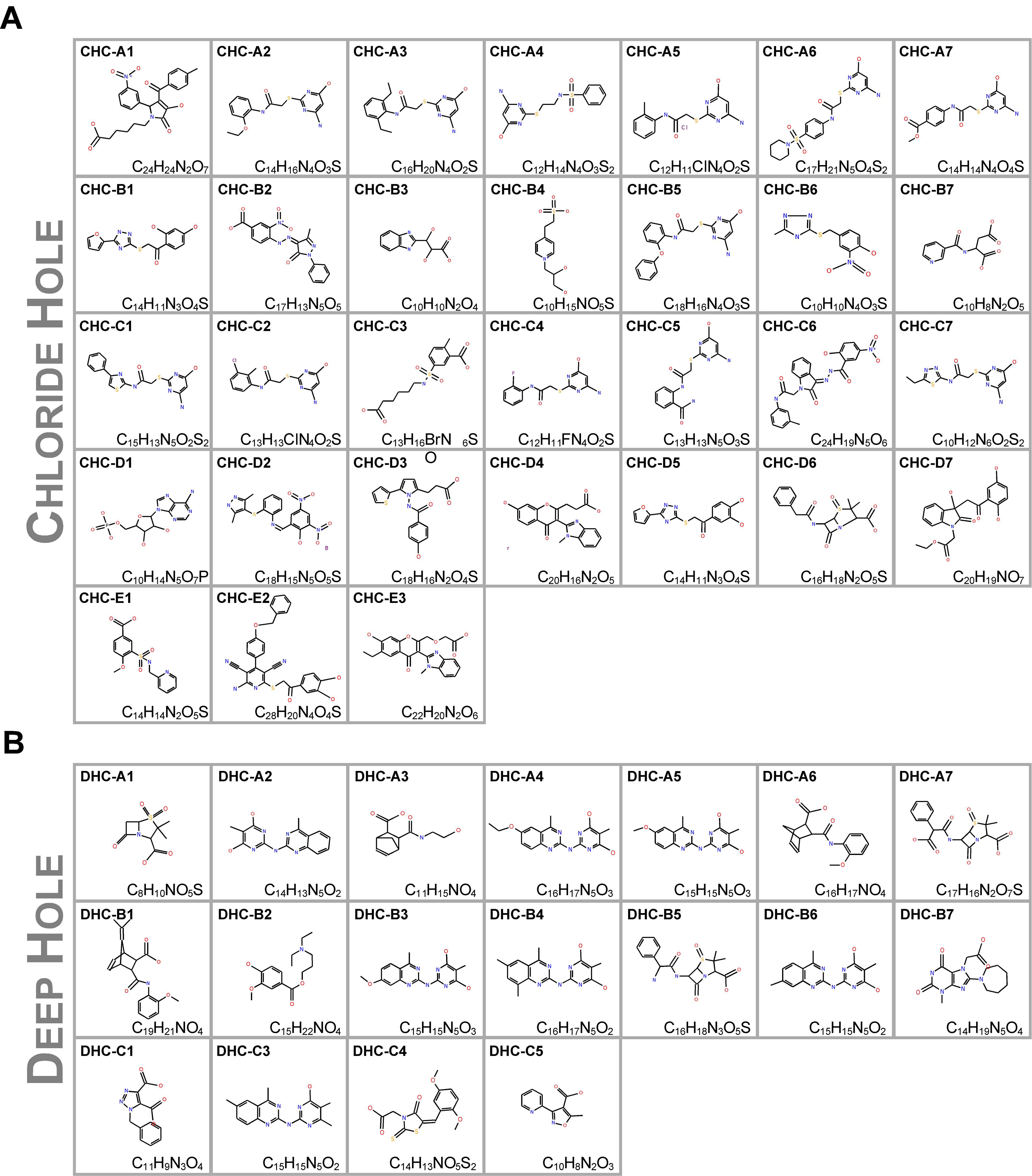

Supplement: Figure S3 — Chloride and deep hole compounds analyzed in HCS assay. Chemical structures and formulas are shown. Abbreviations: CHC, chloride hole compound; DHC, deep hole compound. (TIF) [file pone.0018253.s003.tif]
