# Supplementary material for: Bioassays to Monitor Taspase1 Function for the Identification of Pharmacogenetic Inhibitors
Source: PLoS One. 2011 May 25;6(5):e18253. doi: 10.1371/journal.pone.0018253 (PMC3102056; doi:10.1371/journal.pone.0018253)
Supplement: Table S1 — Oligonucleotides used for PCR amplification and cloning. (DOC) [file pone.0018253.s004.doc]

| **Primer / Oligo** | **Sequence** |
| --- | --- |
| Taspase-NdeI*3 | 5’-GGAATTCCATATGACCATGGAGAAGG-3’ |
| Taspase-XhoI*5 | 5’-CCGCTCGAGGTTCACTGGGCTCTCCA-3’ |
| Taspase-BamHI*3 | 5’-TTTGGATCCACATGACCATGGAGAAGGGGATG-3’ |
| Taspase-NheI*5 | 5’-AAAGCTAGCGTTCACTGGGCTCTCCAGGCGG-3’ |
| Taspase_stop-EcoRI*5 | 5’-TTTGAATTCTTAGTTCACTGGGCTCTCCAGGCGG-3’ |
| mCherry-NheI*3 | 5’-TTTGCTAGCAAGGGCGAGGAGGATAAC-3’ |
| mCherry_stop-EcoRI*5 | 5’-TTTGAATTCTTACTTGTACAGCTCGTCC-3’ |
| mCherry_KpnI*5 | 5’-TTTGGTACCCTTGTACAGCTCGTCCATGC-3’ |
| Puro-NcoI*3 | 5’-AAACAGTTCCGCCCATTCTCCGCCCCATGGCTGAC-3’ |
| Puro-NcoI*5 | 5’-AAACCATGGGTTAGGCACCGGGCTTGCGGGTCATG-3’ |
| 2Cl-EcoRI*3 | 5’-CCGGAATTCCAGGAGGTATCCCCGTCGCAGTGCCCGTGC-3’ |
| 2Cl-XhoI*5 | 5’-CCGCTCGAGAACAGAACTCTTAGTGACATGTTCTTTC-3’ |
| Cl2^+^-NotI*3 | 5’GGCCGCAAAAATCAGCCAGCTTGATGGTGTTGATGATGGCTCTGGCAGCGC-3’ |
| Cl2^+^-XhoI*5 | 5’-TCGAGCGCTGCCAGAGCCATCATCAACACCATCAAGCTGGCTGATTTTTGC-3’ |
| Cl2^+^_K1A*sense | 5’-GGCCGCAGCAATCAGCCAGCTTGATGGTGTTGATGATGGCTCTGGCAGCGC-3’ |
| Cl2^+^_K1A*antisense | 5’-TCGAGCGCTGCCAGAGCCATCATCAACACCATCAAGCTGGCTGATTGCTGC-3’ |
| Cl2^+^_I2A*sense | 5’-GGCCGCAAAAGCCAGCCAGCTTGATGGTGTTGATGATGGCTCTGGCAGCGC-3’ |
| Cl2^+^_I2A*antisense | 5’-TCGAGCGCTGCCAGAGCCATCATCAACACCATCAAGCTGGCTGGCTTTTGC-3’ |
| Cl2^+^_S3A *sense | 5’-GGCCGCAAAAATCGCCCAGCTTGATGGTGTTGATGATGGCTCTGGCAGCGC-3’ |
| Cl2^+^_S3A *antisense | 5’-TCGAGCGCTGCCAGAGCCATCATCAACACCATCAAGCTGGGCGATTTTTGC-3’ |
| Cl2^+^_Q4A*sense | 5’-GGCCGCAAAAATCAGCGCGCTTGATGGTGTTGATGATGGCTCTGGCAGCGC-3’ |
| Cl2^+^_Q4A*antisense | 5’-TCGAGCGCTGCCAGAGCCATCATCAACACCATCAAGCGCGCTGATTTTTGC-3’ |
| Cl2^+^_L5A*sense | 5’-GGCCGCAAAAATCAGCCAGGCTGATGGTGTTGATGATGGCTCTGGCAGCGC-3’ |
| Cl2^+^_L5A*antisense | 5’-TCGAGCGCTGCCAGAGCCATCATCAACACCATCAGCCTGGCTGATTTTTGC-3’ |
| Cl2^+^_D6A*sense | 5’-GGCCGCAAAAATCAGCCAGCTTGCTGGTGTTGATGATGGCTCTGGCAGCGC-3’ |
| Cl2^+^_D6A*antisense | 5’-TCGAGCGCTGCCAGAGCCATCATCAACACCAGCAAGCTGGCTGATTTTTGC-3’ |
| Cl2^+^_G7A*sense | 5’-GGCCGCAAAAATCAGCCAGCTTGATGCTGTTGATGATGGCTCTGGCAGCGC-3’ |
| Cl2^+^_G7A*antisense | 5’-TCGAGCGCTGCCAGAGCCATCATCAACAGCATCAAGCTGGCTGATTTTTGC-3’ |
| Cl2^+^_V8A*sense | 5’-GGCCGCAAAAATCAGCCAGCTTGATGGTGCTGATGATGGCTCTGGCAGCGC-3’ |
| Cl2^+^_V8A*antisense | 5’-TCGAGCGCTGCCAGAGCCATCATCAGCACCATCAAGCTGGCTGATTTTTGC-3’ |
| Cl2^+^_D9A*sense | 5’-GGCCGCAAAAATCAGCCAGCTTGATGGTGTTGCTGATGGCTCTGGCAGCGC-3’ |
| Cl2^+^_D9A*antisense | 5’-TCGAGCGCTGCCAGAGCCATCAGCAACACCATCAAGCTGGCTGATTTTTGC-3’ |
| Cl2^+^_D10A*sense | 5’-GGCCGCAAAAATCAGCCAGCTTGATGGTGTTGATGCTGGCTCTGGCAGCGC-3’ |
| Cl2^+^_D10A*antisense | 5’-TCGAGCGCTGCCAGAGCCAGCATCAACACCATCAAGCTGGCTGATTTTTGC-3’ |
| Cl2^+^_Q4N*sense | 5’-GGCCGCTAAGATCAGCAATCTTGATGGTGTTGATGATGGCTCTGGCAGCGC-3’ |
| Cl2^+^_Q4N*antisense | 5’-TCGAGCGCTGCCAGAGCCATCATCAACACCATCAAGATTGCTGATCTTAGC-3’ |
| Cl2^+^_L5F*sense | 5’-GGCCGCAAAAATCAGCCAGTTCGATGGTGTTGATGATGGCTCTGGCAGCGC-3’ |
| Cl2^+^_L5F*antisense | 5’-TCGAGCGCTGCCAGAGCCATCATCAACACCATCGAACTGGCTGATTTTTGC-3’ |
| Cl2^+^_L5I*sense | 5’-GGCCGCTAAGATCAGCCAGATCGATGGTGTTGATGATGGCTCTGGCAGCGC-3’ |
| Cl2^+^_L5I*antisense | 5’-TCGAGCGCTGCCAGAGCCATCATCAACACCATCGATCTGGCTGATCTTAGC-3’ |
| Cl2^+^_L5Q*sense | 5’-GGCCGCAAAAATCAGCCAGCAAGATGGTGTTGATGATGGCTCTGGCAGCGC-3’ |
| Cl2^+^_L5Q*antisense | 5’-TCGAGCGCTGCCAGAGCCATCATCAACACCATCTTGCTGGCTGATTTTTGC-3’ |
| Cl2^+^_L5W*sense | 5’-GGCCGCAAAAATCAGCCAGTGGGATGGTGTTGATGATGGCTCTGGCAGCGC-3’ |
| Cl2^+^_L5W*antisense | 5’-TCGAGCGCTGCCAGAGCCATCATCAACACCATCCCACTGGCTGATTTTTGC-3’ |
| Cl2^+^_L5Y*sense | 5’-GGCCGCAAAAATCAGCCAGTATGATGGTGTTGATGATGGCTCTGGCAGCGC-3’ |
| Cl2^+^_L5Y*antisense | 5’-TCGAGCGCTGCCAGAGCCATCATCAACACCATCATACTGGCTGATTTTTGC-3’ |
| Cl2^+^_D9E*sense | 5’-GGCCGCTAAGATCAGCCAGCTTGATGGTGTTGAAGATGGCTCTGGCAGCGC-3’ |
| Cl2^+^_D9E*antisense | 5’-TCGAGCGCTGCCAGAGCCATCTTCAACACCATCAAGCTGGCTGATCTTAGC-3’ |
| Cl2^+^_D10E*sense | 5’-GGCCGCTAAGATCAGCCAGCTTGATGGTGTTGATGAAGGCTCTGGCAGCGC-3’ |
| Cl2^+^_D10E*antisense | 5’-TCGAGCGCTGCCAGAGCCTTCATCAACACCATCAAGCTGGCTGATCTTAGC-3’ |
| TF2A-NotI*3 | 5’-TTTGCGGCCGCAATGGCGAACTCGGCAAATAC-3’ |
| TF2A-XhoI*5 | 5’-AAACTCGAGCCCATTCTGCATCTCCAATG-3’ |
| TF2A-BamHI*3 | 5’-TTTGGATCCACATGGCGAACTCGGCAAATAC-3’ |
| TF2A-NheI*5 | 5’-TTTGCTAGCCCATTCTGCATCTCCAATG-3’ |
| DPLOZ-NotI*3 | 5’-tttGCggccgcgTGCAGAAATACCCACAG-3’ |
| DPLOZ-XhoI*5 | 5’-aaaCTCGAGaAGCATCTTTGTGAAAGATGG-3’ |
| FRM4B-NotI*3 | 5’-tttGCggccgcgCTGTCGGATTACGAGACTC-3’ |
| FRM4B -XhoI*5 | 5’-aaaCTCGAGaGACTAATGTTCCAGGCTTTG-3’ |
| PTRZ-NotI*3 | 5’-tttGCggccgcgCGAGTCGTTTATGATACC-3’ |
| PTRZ-XhoI*5 | 5’-aaaCTCGAGaGCATATGGCTACTATCTGAAG-3’ |
