# Supplementary material for: Bioassays to Monitor Taspase1 Function for the Identification of Pharmacogenetic Inhibitors
Source: PLoS One. 2011 May 25;6(5):e18253. doi: 10.1371/journal.pone.0018253 (PMC3102056; doi:10.1371/journal.pone.0018253)
Supplement: Table S2 — Taspase1 expression levels and proteolytic activity in solid cancer cell line models. (DOC) [file pone.0018253.s005.doc]

| **Cell line** | **Origin** | **Taspase1 protein level** | **Cleavage of TS-Cl2^+^** |
| --- | --- | --- | --- |
| 293T | human embryonal kidney (mesenchymal) | - | - |
| A431 | human epidermoid carcinoma (epithelial) | - | - |
| RKO | human colon carcinoma (epithelial) | - | - |
| SaOs | human osteogenic sarcoma (epithelial) | +++ | +++ |
| SW480 | human colon adenocarcinoma (epithelial) | ++ | + |

-: not detectable, ++: intermediate, +++: high.
