# Supplementary material for: Bioassays to Monitor Taspase1 Function for the Identification of Pharmacogenetic Inhibitors
Source: PLoS One. 2011 May 25;6(5):e18253. doi: 10.1371/journal.pone.0018253 (PMC3102056; doi:10.1371/journal.pone.0018253)
Supplement: Table S3 — List of potential human Taspase1 targets predicted by ScanProsite . (DOC) [file pone.0018253.s006.doc]

**Supplementary Table S3 - List of potential human Taspase1 targets predicted by *ScanProsite***

Gene loci, SwissProt accession numbers, gene names and aliases, as well as number of aa are shown. Sequence: aa containing the predicted Taspase1 cleavage site(s) (consensus marked in bold).

| **Gene Locus** | **Swiss Prot** | **Alias** | **Number of aa** | **Sequence** |
| --- | --- | --- | --- | --- |
| MLL1 | Q03164 | MLL, HRX, ALL1 | 3969 | SAEG **QVDGADD** LSTS |
|  |  |  |  | PKIS **QLDGVDD** GTES |
| MLL4 | Q9UMN6 | MLL2, MLL4, HRX2, TRX2 | 2715 | PRIE **QLDGVDD** GTDS |
| MYO1F | O00160 | MYO1F, Myosin-le, myosin-ID | 1098 | SDTY **QVDGTDD** RSDF |
| IPPK | Q9H8X2 | IPPK, IPK1, IP5K, INSP5K2 | 491 | RKTL **QIDGP**Y**D** EAFY |
| NXF2 | Q9GZY0 | NXF2, CT39, TAPL2 | 626 | NKLY **QLDGL**S**D** ITEK |
| NXF5 | Q9H1B4 | NXF5, TAPL1 | 397 | NKLY **QLDGL**S**D** ITEK |
| RBGP1 | Q9Y3P9 | RABGAP1, GAPCenA, TBC1D11 | 1069 | VKRS **QLDGE**G**D** GPLS |
| USF2 | Q15853 | USF2, FIP | 346 | VTDG **QLDGQ**G**D** TAGA |
| PRS4 | P62191 | PSMC1, S4, p56, P26S4 | 440 | ELLN **QLDGFD**S RGDV |
| PRS7 | P35998 | PSMC2, MSS1, S7 | 433 | ELIN **QLDGFD**P RGNI |
| PTPRG | P23470 | PTPRG, R-PTP-GAMMA, PTPG | 1445 | YQEL **QLDGFD**N ESSN |
| PTRZ | P23471 | PTPRZ1, PTP18, HPTPzeta | 2315 | VLYQ **QLDGED**Q TKHE |
| SDCB2 | Q9H190 | SDCBP2, SITAC18 | 292 | DQLL **QIDGRD**C AGWS |
| SDCG1 | O60524 | SDCCAG1, NY-CO-1 | 1076 | EEME **QLDGGD**T SSDE |
| TF2AA | P52655 | GT2A1, TF2A, TFIIA | 485 | GEII **QVDGSGD** TSSN |
| ATP9A | O75110 | ATP9A, ATPIIA | 1047 | LRTD **QLDGE**T**D** WKLR |
| ATP9B | O43861 | ATP9B, NEO1L, HUSSY-20 | 1147 | IRTD **QLDGE**T**D** WKLK |
| DPOLZ | O60673 | REV3L, POLZ | 3130 | LSIP **QLDGT**A**D** ENSD |
| GGT5 | P36269 | GGT5, GGT-REL, GGTLA1 | 586 | LIRQ **QIDGR**G**D** HQLS |
| HOOK1 | Q9UJC3 | HOOK1, HK1 | 728 | EKLD **QLDGS**F**D** DPNT |
| ABCA1 | O95477 | ABCA1, TGD, ABC1, CERP | 2261 | FWEQ **QLDGLD**W TAQD |
| ARSJ | Q5FYB0 | ARSJ, ASJ | 599 | DEDI **QLDGYD**I WETI |
| FRM4B | Q9Y2L6 | FRMD4B, GRSP1 | 980 | TEIS **QLDGTD**G NQLE |
| PDZD6 | Q9ULD6 | INTU, PDZD6, PDZK6 | 942 | TTLH **QLDGVD**S RIDE |
| POLN | P29324 | POLN | 1693 | VKVS **QVDGR**I**D** CETL |
